# Genetic diversity and silencing suppression effects of *Rice yellow mottle virus *and the P1 protein

**DOI:** 10.1186/1743-422X-5-55

**Published:** 2008-04-30

**Authors:** Christelle Siré, Martine Bangratz-Reyser, Denis Fargette, Christophe Brugidou

**Affiliations:** 1 Institut de Recherche pour le Développement (IRD), UMR LGDP, 34394 Montpellier Cedex 5, France; 2 Institut de Recherche pour le Développement (IRD), UMR RPB, 34394 Montpellier Cedex 5, France

## Abstract

**Background:**

PTGS (post-transcriptional gene silencing) is used to counter pathogenic invasions, particularly viruses. In return, many plant viruses produce proteins which suppress silencing directed against their RNA. The diversity of silencing suppression at the species level in natural hosts is unknown.

**Results:**

We investigated the functional diversity of silencing suppression among isolates of the African RYMV (*Rice yellow mottle virus*) in rice. The RYMV-P1 protein is responsible for cell-to-cell movement and is a silencing suppressor. Transgenic *gus*-silencing rice lines were used to investigate intra-specific and serogroup silencing suppression diversity at two different levels: that of the virion and the P1 silencing suppressor protein. Our data provide evidence that silencing suppression is a universal phenomenon for RYMV species. However, we found considerable diversity in their ability to suppress silencing which was not linked to RYMV phylogeny, or pathogenicity. At the level of the silencing suppressor P1 alone, we found similar results to those previously found at the virion level. In addition, we showed that cell-to-cell movement of P1 was crucial for the efficiency of silencing suppression. Mutagenesis of P1 demonstrated a strong link between some amino acids and silencing suppression features with, one on the hand, the conserved amino acids C95 and C64 involved in cell-to-cell movement and the strength of suppression, respectively, and on the other hand, the non conserved F88 was involved in the strength of silencing suppression.

**Conclusion:**

We demonstrated that intra-species diversity of silencing suppression is highly variable and by mutagenesis of P1 we established the first link between silencing suppression and genetic diversity. These results are potentially important for understanding virus-host interactions.

## Background

PTGS (post-transcriptional gene silencing) is conserved among eukaryote kingdoms and is a gene-regulatory mechanism involved in several control processes, including development, maintenance of genome stability, and defence against invasive pathogens. This molecular mechanism is initiated by double-stranded RNA (dsRNA) molecules, or RNA with secondary structures, and results in a reduced steady-state level of cognate cytoplasmic mRNA [1,2], in which small RNAs (21–24 nucleotides) play crucial roles. Among these small RNAs, two functionally different RNAs, microRNAs (miRNA) and small-interfering RNAs (siRNA), have been characterized [3]. RNA-dependent RNA polymerase activity (RdRP) leads to production of dsRNAs molecules that are recognized and cleaved through the sclicer activity of RNAseIII (DICER-like protein). Thus small RNAs are loaded to RNA Induced silencing complex (RISC) and served as guides to cleave cognate mRNAs. It has been well described that PTGS is a major defence response against viruses [4]. Hence, viruses have been described as activators, as well as, targets of this mechanism [5-7]. To facilitate their replication and movement among host cells, plant viruses have acquired mechanisms to suppress gene silencing targeted at their RNA [8,9]. Such proteins are usually involved in viral pathogenicity [9] and also in virus spread, in cell-to-cell as well as in long-distance movement [10]. Many proteins that prevent, or suppress, the silencing of viral RNA have now been identified. These counter-defensive proteins can act at different steps in the PTGS pathway, e.g., involving the silencing signal itself or its subsequent propagation [8,11]. There is considerable diversity in the sequences of these suppressor proteins, as well as in their targets and modes of action [12-14]. This suggests that viruses have no general strategy for suppressing the silencing of their RNA.

The *Rice yellow mottle virus*, belonging to the *Sobemovirus *genus, is endemic to Africa and is the major pathogen of irrigated rice [15,16]. Its genome and particle structure have been described [17,18]. P1 is a 18–19 kDa protein encoded by the first open reading frame (ORF) of RYMV positive sense single-stranded RNA, which contains four partially overlapping ORFs [19]. With up to 17.8% amino acid sequence divergence, the genetic diversity of P1 protein is higher than for other viral proteins [19]. This protein is required for viral replication and cell-to-cell movement [20]. It also acts as a non-autonomous-cell silencing suppressor in *Nicotiana benthamiana *[9,21].

It is well known that viral suppressors differ considerably in their structure and function [22]. However, at the virus species level, functional diversity of these proteins is unknown. In addition, nothing is known at the viral level as to how they suppress the silencing of their RNA during the infection of a natural host. We investigated both of these aspects using RYMV and P1 protein as model systems, to study the functional diversity of silencing suppression from various virus isolates. In this paper, we analysed silencing suppression of RYMV in its natural host at the virion and P1 protein levels. Such an analysis was possible since genetic diversity of the RYMV is well characterised [19,23,24] and its phylogeography could be reconstructed in detail to highlight links between the geographical and ecological origins of virus isolates [19,25]. Using a transgenic rice line, containing a silenced β-glucoronidase (GUS) transgene (*iudA*), we investigated by GUS activity reversion the functional diversity of PTGS suppression upon virion inoculation or ectopic P1 gene expression. We thus were able to demonstrate for the first time the high diversity of silencing suppression at the intra-species level, suggesting a complex mechanism in silencing suppression at the virus scale. Using P1 mutants we found that cell-to-cell movement and the efficiency of silencing might be linked with variation in the efficiency of silencing suppression.

## Methods

### Plant material

**Figure 1:**
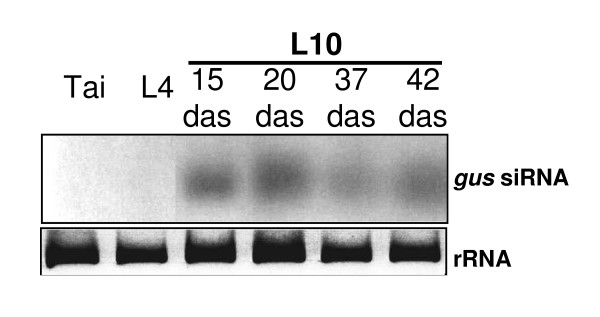
**Assessment of the PTGS mechanism with *gus*-specific siRNA detection by Northern blot in different rice varieties at different stages of plant development**. The rice plants used were Tai; non transgenic cv. Taipei, L4; a transgenic *gus*-expressing line, L10; a *gus*-silenced transgenic line revealed by histochemical staining collected at 15, 20, 37 and 42 days after seeding (das). EtBr staining of rRNA served as a loading control.

Transgenic *O. sativa *L. (*O. s*.) spp. *japonica *cv. Nipponbare containing the *uidA (gusA) *transgene from pCAMBIA 1301 (GenBank accession No. AF234297) and carrying a single copy of T-DNA [26] (C. Sallaud, unpublished data) were used. Transgenic line L4 constitutively expresses the *uidA *gene and the transgenic line L10 is *gus*-silenced (Figure 1). L10 expressed a PTGS phenotype for expression of the *uidA *gene on the basis of GUS staining in leaves or roots. With *gus*-specific siRNA detection, we correlated the L10-phenotype to PTGS and demonstrated its stability over the lifetime of vegetative plants from germination (Figure 1). L10 was used as a model in our silencing suppression studies during host-pathogen interactions. Plants were grown and maintained in controlled conditions in a confined greenhouse (CGG consent for GMO culture n° 3576) under 12 h light at 28°C, 12 h dark at 24°C and at a relative humidity of 70%. As a control, wild-type *O. sativa *spp. *japonica *cv. Taipei (Tai.) was used.

### Virus isolates and inoculation

**Figure 2:**
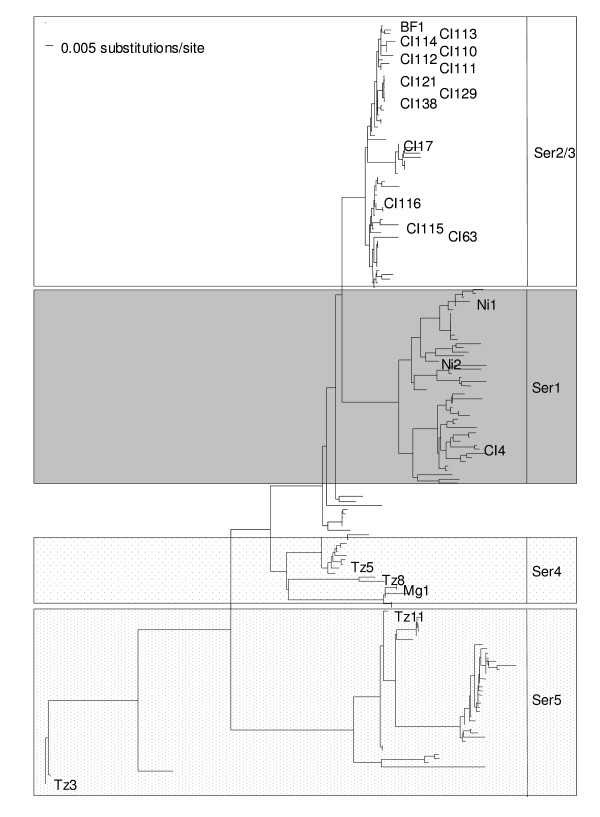
**ORF4 (CP)-based phylogenetic tree**. Phylogenetic tree reconstructed from 179 partially sequenced isolates from the 15 countries where RYMV has been reported: Benin, Burkina Faso (BF), Chad, Ivory Coast (CI), Cameroon, Ghana, Guinea, Kenya, Madagascar (Mg), Mali, Niger, Nigeria (Ni), Sierra Leone, Tanzania (Tz) and Togo. The Kimura2 substitution model with estimates of the alpha parameter of the gamma shape was applied. The serotype of the isolates defined by reactions with a set of monoclonal antibodies in ELISA tests (47) is indicated (by vertical bars) at the right of the figure.

We used 10 fully sequenced RYMV isolates belonging to different serogroups and representative of viral diversity in Africa (i.e. with 9.9% maximum diversity) [19,23], these were; BF1, CI63, CI4, Ni1, Ni2, Mg1, Tz3, Tz5, Tz8 and Tz11. There was a second set of 10 isolates belonging to serogroup 2, showing variable pathogenicity assessed through symptom intensity, but low sequence diversity (2%). These were; CI110, 111, 112, 113, 114, 115, 116, 121, 129, and 138 [27] (Figure 2). They were independently propagated in the susceptible cultivar *O. sativa *spp. *indica *cv. IR64.

Two weeks after seeding, plants (2 leaves) were mechanically inoculated as described elsewhere [28] with diluted sap (1/10 w/v). Each isolate was independently inoculated into 20 plants that were grown separately to limit cross contamination. Viral inoculum were adjusted to a standard concentration, as estimated by DAS-ELISA assays, to prevent variation in silencing suppression due to differences in initial virus delivery. In order to avoid bias due to inoculation heterogeneity, leaf samples were collected from each inoculated plant at 35 or 40 dpi and separated into two independent samples before being ground for proteins (fluorimetry, ELISA and western) and RNA extractions.

### Plasmid construction and biolistic delivery experiment

P1 sequences with UTR (from 1 to 709 nt) [17] from five isolates representative of the diversity of P1 with a divergence up to 17.8% were selected. These isolates are representative of the diversity of the other parts of the genome as phylogenetic reconstruction from the P1, the CP and the full genome are congruent. They were cloned into the CaMV 35S constitutive promoter and terminator, that were named sTz3; sMg1; sCI63; sBF1; sTz8. PCR fragments of P1 sequences (underlined) were amplified with: forward GAATTC**AAGCTT**GACAATTGAAGCTAGGAAAGGAGC, reverse GAATTC**TCTAGA**CGCGGCC GCTATCAA, and cloned into the XbaI/HindIII (in bold) restriction site from the 35S cassette. These constructs were delivered twice onto pieces of L10 leaves (collected 2 weeks after seedling) by particle bombardment. For each P1 tested, 20 leaves were cut into 10 cm strips. In order to detect the specific effect of P1 and to avoid potentially unfavourable viral translation, we analysed leaves at 2 days post-delivery (dpd). Histological experiments were performed in the presence of ferro- and ferri-cyanide, so as to ensure wide range spread of coloration was not influenced by stain spreading and only reflected presence of GUS activity.

#### Biolistic delivery

Five micrograms of sP1s were inoculated onto pieces of leaves from the L10 *gus*-silenced line by biolistic delivery (Biorad Biolistic PDS 1000/He) after 3 h incubation in MES buffer containing 10% (w/v) sucrose. Leaves were bombarded at a pressure of 1100 psi and a flight distance of 6 cm with tungsten-coated particles. Each construct was delivered to 20 leaf pieces that were bulked for analysis at the end of the experiment. Leaves were then placed at 26°C for 2 days in 16 h light, 8 h dark at a relative humidity of 60%, on 1.5% phytagel and 0.5 w/v MES containing 3% (w/v) sucrose.

### RNA and protein analyses

Total RNA was extracted from rice leaves with TRIzol reagent (Invitrogen™) according to the manufacturers' instructions. Analysis of low molecular weight RNA was performed on 15 μg total RNA as described previously [29]. Analysis of mRNA was performed with NorthernMax^®^-Gly (Ambion^®^) as recommended by the manufacturer. Radio-labelling of the probe and hybridization was performed as done in ref. [30]. A 808 bp PCR amplified fragment (808 to 1616 bp) of *GusA *was used for the specific probe preparation. Radio-labelled signals were detected either by autoradiography or by scanning with Typhoon (Amersham). RNA band intensities were quantified using Image Quant software (Molecular Dynamics).

Soluble proteins were extracted from rice leaves in a sodium phosphate buffer (50 mM NaHPO_4_, 10 mM Na_2_EDTA, 1 mg.ml^-1 ^N-Laurylsarcosine, 0.1% v/v Triton ×100) by two successive centrifugations of 20000 g at 4°C. The Bradford colorimetric method (Coomassie protein assay kit, Pierce) [31] was used for protein quantification. Twenty micrograms of protein were resolved by SDS-PAGE and transferred by electroblotting onto a nitrocellulose membrane (Trans-Blot^® ^Transfer Medium, Biorad). A diluted rabbit polyclonal antibody directed against the P1 protein (1/500) was used and revealed by a second (1/40000), horseradish peroxidase-conjugate (Pierce) through Supersignal^® ^West-Pico chemiluminescence substrate. Blots were exposed to CL-XPosure™ film (Pierce).

### DAS ELISA assays

The virus concentration was evaluated by DAS-ELISA as described previously [32]. DAS-ELISA was performed with diluted (1/1000 v/v) polyclonal antiserum against an isolate from Madagascar (RYMV-Mg). Positive reactions were detected after incubation with alkaline phophatase-conjugated polyclonal antibody to RYMV and substrate, with absorbance read using a Multiskan fluorimeter (Labsystem).

### GUS assays

Histochemical staining for GUS activity and GUS assays were performed according to Jefferson [33] and involved pieces of leaves randomly collected from two to five plants at 1 and 2 dpi (inoculated leaves), and 7, 14, 35 and 40 dpi (systemically infected leaves).

As described previously [34], proteins were incubated with 1 mM of 4-methylumbelliferyl-β-D-glucuronide (MUG, Sigma^®^). Fluorescence was measured at 15 min intervals for 2 h with a Fluoroskan fluorimeter (Labsystem), with a 365 nm excitation filter and a 455 nm emission filter. Calibration was performed with quantification of fluorescence of soluble 4-methylumbelliferone (MU, Sigma^®^).

## Results

### Silencing suppression diversity at the RYMV species level

**Figure 3:**
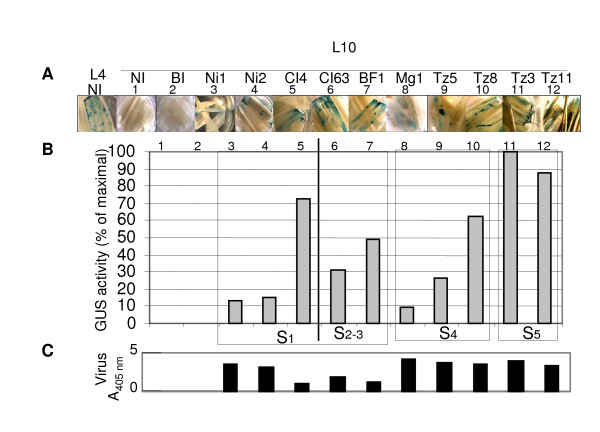
**Heterogeneous silencing suppression caused by highly diverse isolates**. (A) GUS histochemical staining analysis of *gus*-silenced transgenic L10 leaves harvested at 35 dpi, controls correspond to non-inoculated (NI) and buffer-inoculated (BI) leaves from transgenic *gus*-expressing L4 and *gus*-silenced L10. In boxes, isolates belonging to the same serogroup (S, Figure 2) are compared. (B) GUS activity is measured by fluorimetry and is expressed as a percentage of maximal activity measured in Tz3 inoculated samples. (C) ELISA-based measurement of virus content and western blot analysis of P1 protein.

We investigated the diversity of RYMV efficiency in silencing suppression and used a transgenic *gus*-silenced rice line (L10). As seen in Figure 1, silencing of the *gus *transgene, leads to an absence of GUS activity, and is accompanied with a strong accumulation of *gus *specific siRNA. This accumulation is maintained during the life-cycle of rice plants (Figure 1). Moreover, infection with the RYMV virus leads to a restoration of GUS activity concomitant with a decrease of gus-siRNA accumulation. Assuming that silencing suppression activity of RYMV involves restoration of the GUS phenotype and a decrease of steady-state levels of gus-siRNA, we quantified silencing suppression efficiency by evaluating the decrease in steady state levels of siRNA. We separately inoculated 10 RYMV isolates that differed in their coat protein (CP) (Figure 2). From 1 dpi, we revealed GUS reversion for all isolates by observing the blue histochemical staining patterns in both L10-infected leaves and positive controls (i.e. transgenic L4 lines that constitutively expresses the *uidA *transgene) whereas in non-inoculated (NI) and mock-inoculated (BI) L10 leaves, GUS activity was undetectable (Figure 3A) indicating that the reversion of GUS activity in L10-inoculated plants was only due to RYMV and not to mechanical stress caused by the inoculation.

Because histochemical staining of GUS is not a quantitative method the reversion of *uid*A gene expression in L10 was assayed by fluorimetric quantification of GUS activity, in systemically infected leaves. We found that RYMV isolates differed in their efficiency to reverse the constitutive silencing, as illustrated by the heterogeneity of restored GUS activity (Figure 3B). In each serogroup (Figure 3B), we detected a contrasting pattern of reactivation for the *uidA *transgene. In S1, the isolates CI4, Ni1 and Ni2 exhibited distinct effects on silencing suppression (Figure 3B, lanes 3–5). Similarly, the S4 Mg1 isolate weakly suppressed silencing, whereas a strong effect was obtained for the Tz8 isolate (Figure 3B, lanes 8, 10). Finally, strong suppression leading to higher GUS activity was detected for the Tz3 and Tz11 isolates belonging to the S5 serogroup (Figure 3B, lanes 11–12). This result showed that the ability to suppress PTGS was not correlated with the viral phylogeny.

Moreover, we found that viral content was not correlated with GUS activity, as illustrated by isolates belonging to the same serotype Ni2 and CI4, or, CI63 and BF1 isolates (Figure 3C, lanes 3 and 4 or 4 and 5). We also noted that the diversity of silencing suppression displayed by these RYMV isolates was not correlated with viral pathogenicity (data not shown). Indeed, the most pathogenic isolate, BF1, was not the strongest in suppressing PTGS.

Taken together, these results highlighted the prevalence of silencing suppression within the species and showed that *gus*-silenced gene reversion was dependent on viral isolate.

### Silencing suppression diversity at the RYMV serogroup level

As plants infected with different RYMV isolates exhibited variable GUS activity, we investigated the diversity of this restoration at the serogroup level. Thus we considered isolates from the Ivory Coast belonging to the least variable RYMV serogroup S2 (diversity around 2% based on CP sequence) (Figure 2). Ten representative isolates of S2 were chosen for homogeneous biological characters (same collection date and geographical localisation) and were inoculated into L10 plants.

**Figure 4:**
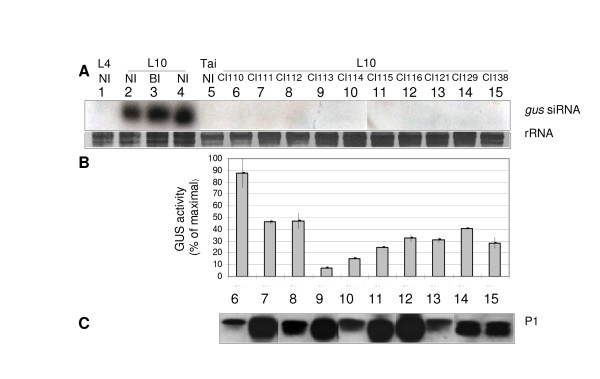
**Heterogeneous silencing suppression caused by isolates from a homogeneous phylogenetic group (i.e. S2 isolates), at 40 dpi**. (A) Northern blot analysis of *gus*-siRNA. Controls group together: positive controls for *gus*-siRNA detection corresponding to RNA from L10 either NI (non-inoculated) or BI (buffer-inoculated) and RNA from non transgenic Tai, or transgenic L4 plants as negative controls. EtBr staining of rRNA served as a loading control. (B) GUS activity is measured by fluorimetry and is expressed as a percentage of the maximal activity measured in CI110 inoculated plants. Analyses were carried out with soluble protein extracted from L10 leaves infected by different isolates. Data represent average values of two independent measurements with standard deviations indicated. (C) Western blot analysis of P1 protein.

Silencing suppression showed by the restoration of GUS activity was followed by histochemical staining of GUS at 1, 2, 7, 15, 17, 21, 27, 31, and 40 dpi. At 40 dpi, steady-state siRNA levels were monitored with Northern blots. *Gus*-specific siRNA were not detected in systemically infected leaves in contrast to NI or BI L10 leaves (Figure 4A). This revealed that *uid*A gene silencing had been reverted by all S2 isolates tested. To analyze more closely the qualitative effect of the different isolates, we compared GUS activity levels measured by fluorimetry (Figure 4B) to viral amounts measured by ELISA (Table 1) in systematically infected leaves. An analysis of covariance revealed that at 40 dpi a large part of the variability in silencing suppression was due to an isolate effect, although part of the reversion was due to an effect of virus content (Table 1). We thus revealed highly heterogeneous responses in GUS activity reversion following viral infection (Figure 4B). Isolate CI110, is considered to be strong suppressor of constitutive silencing in the *iud*A gene, (Figure 4B). The weaker suppressors were isolates CI113 and 114, with less than 20% maximal activity restoration (Figure 4B). Western blot analyses showed that the efficiency of silencing suppression was not correlated with RYMV-P1 protein suppressor accumulation in leaves (Figure 4C). As all isolates belong to the same serogroup, this quantitative variation of P1 accumulation should not be linked to polyclonal antibody affinity. In addition, sequences analysis of P1 proteins found no obvious correlation between the efficiency of silencing suppression and genetic diversity of the P1 protein (data not shown) suggesting that silencing suppression when analyzed at the virion scale is more complex than previously thought. As noted above, the efficiency of silencing suppression is not correlated to symptoms expressed by these isolates (data not shown).

**Table 1:** Virus content and restoration of GUS activity

	Gus activity	A_405 nm_
CI110-1	80	0,53
CI110-2	60,4	0,14
CI111-1	38,5	0,62
CI111-2	35,7	0,49
CI112-1	32,2	0,22
CI112-2	42,8	0,26
CI113-1	5,8	0,48
CI113-2	5,6	0,49
CI114-1	12,3	0,39
CI114-2	12,6	0,27
CI115-1	20	0,54
CI116-1	23,9	0,84
CI116-2	28,4	0,66
CI121-1	23,5	0,27
CI121-2	26,6	0,55
CI129-1	33,1	0,58
CI129-2	31,8	0,29
CI138-1	26,6	0,31
CI138-2	19	0,23

	**F. ratio**	**p**

**Virus content effect**	**8.97**	**0.02**

**Isolate effect**	**33.89**	**0.00002**

Effect of virus content expressed in absorbance units read at 405 nm, on restoration of GUS activity measured by fluorimetry and expressed in pmol MU/min/μg protein. Results of covariance analyses to evaluate both virus content and isolate effects with the Fisher test.

Taken together our results at the virion scale demonstrate that the accumulation and genetic diversity of the P1 suppressor protein were not correlated to heterogeneity in the reversion of GUS activity that we observed.

### Effect of ectopic P1 protein expression on silencing suppression

As the effect of RYMV on *uidA *silencing suppression was not linked to viral diversity, pathogenicity or P1 accumulation, we attempted to assess the particular effect of the P1 protein identified as the RYMV silencing suppressor protein [9]. Different P1s representative of the viral diversity were cloned under control of the CaMV 35S promoter. This was done in order to determine whether the sequence diversity of this suppressor reflects functional diversity and to accurately determine the qualitative effect of the P1 protein on silencing suppression.

**Figure 5:**
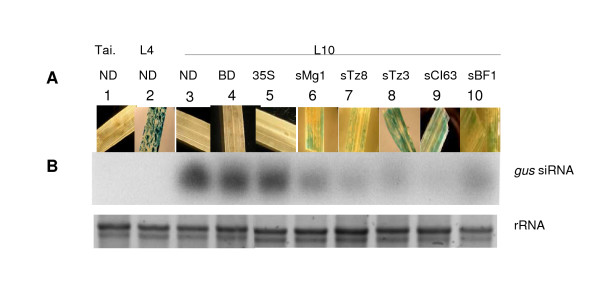
**Effect of the P1 protein from different RYMV isolates on silencing suppression in rice**. Transgenic L10 leaves were analysed after different treatments; non-biolistic delivery (ND), buffer delivery (BD), biolistic delivery with a empty 35S vector (35S), or vectors containing different sP1 from RYMV isolates representative of the viral phylogeny (CI63, Mg1, Tz8, Tz3, BF1). Non transgenic Tai and transgenic L4 served as controls. (A) Photographs correspond to GUS staining at 2 dpd of inoculated leaves. (B) Quantitative effect of different P1 at 2 dpd on *gus*-specific siRNA with Northern blot experiments. EtBr staining of rRNA served as a loading control.

We demonstrated a lack of effect due to biolistic delivery and the expression vector by using empty 35S (Figure 5A and 5B lane 5). At 1 day post delivery (dpd), we revealed the reversion of GUS activity, except for sTz8 for which GUS activity was only observed at 2 dpd (Figure 5A). We thus demonstrated for the first time in a natural host, that all P1s from different RYMV isolates were silencing suppressors. In addition, we observed the reversion of GUS activity over the entire leaf, even where the P1 protein had not been delivered, suggesting strong movement of this protein (Figure 5A).

To further examine whether P1 acts differently in relation to variation in its sequence, we evaluated the steady-state level of *gus*-specific siRNA by Northern blot. Every P1 induced a decrease in the steady-state level of *gus*-siRNA, as compared to siRNA detected in L10 controls (ND, BD and 35S) (Figure 5B). Moreover, as shown in Figure 5B, the different P1s exhibited contrasting activity in silencing suppression, as revealed by the different siRNA patterns. These results highlight the qualitative effect of different P1s and that they could be grouped into two classes according to their efficiency of suppression. Indeed, we observed that GUS activity restoration with sCI63, sTz3 and Tz8 was accompanied by a dramatic decrease in siRNA levels. Consequently, we concluded these proteins are highly efficient in transgene-induced silencing suppression in rice. Conversely, in case of sBF1 and sMg1, we observed a slight decrease in siRNA levels (Figure 5B), and they were thus classified as weak silencing suppressors.

Furthermore, as previously demonstrated at the virion scale, the efficiency of these silencing suppressors is not correlated with viral phylogeny. Indeed, according to RYMV evolutionary history [19], P1s from isolates belonging to the same phylogenetic group can be either strong (sCI63) or weak silencing suppressors (sBF1) (Figure 2 and 5B). In addition, P1s from distant isolates were similar in their silencing suppression efficiencies, as illustrated by the weak suppressors sBF1 and sMg1 (Figure 2 and 5B).

### Movement of P1 protein and silencing suppression

**Figure 6:**
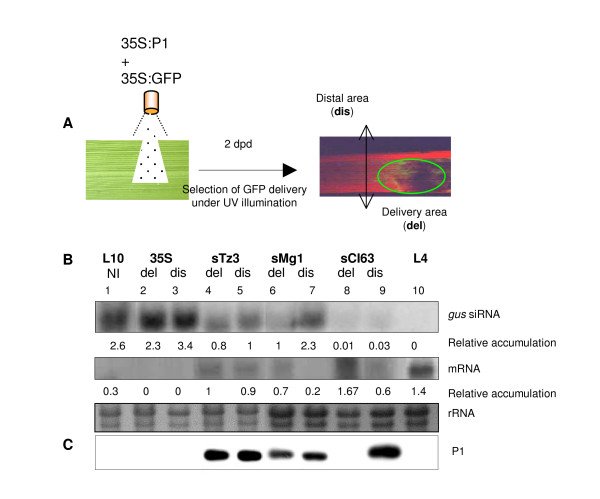
**Movement of P1 protein and silencing suppression**. (A) Schematic representation of the experimental design to distinguish delivery (del) and distal (dis) points after bombardment. (B) Northern analysis of *gus*-siRNA and mRNA at 2 days post delivery (dpd) with sTz3, Mg1 and CI63 P1s in the del or dis parts of the leaf. 35S is an empty cassette (negative control) and EtBr staining of rRNA served as a loading control. (C) Western blot analysis of P1 protein. Relative accumulation to the loading control are shown, RNA band intensities were quantified using Image Quant software (Molecular Dynamics).

As we found a qualitative effect of the different P1s, and as it has been demonstrated that silencing suppression and cell-to cell movement of *Potato potexvirus *X (PVX) are linked [35], we investigated the involvement of cell-to-cell movement of P1 on the qualitative variation in silencing suppression (Figure 6A). We carried out an experiment to analyze P1 protein accumulation at the site of delivery (del) and in distal (dis) areas of L10-inoculated leaves. To do so, we co-bombarded 20 pieces of L10 leaves with every sP1 construct described above and associated with a 35S-GFP plasmid. Under UV illumination at 2 dpd, we were able to discriminate the delivery area from the distal area by GFP fluorescence. We could detect green areas corresponding to the delivery zone (del) and red areas due to chlorophyll fluorescence corresponding to the distal zone (dis) (Figure 6A). To limit any contamination by P1 outside from the delivery zone, we enlarge the delivery zone by an additional 1.5 cm which leads, in addition to a 6 cm flight distance, to an incompatible zone to receive any particles.

We compared the steady-state level of *gus*-siRNA and mRNA, in the delivery and distal areas for each sP1 protein previously selected for a range of variation in their efficiency of silencing suppression (Figure 6B). For the sTz3 protein, accumulation of *gus*-siRNA and mRNA were similar in del and dis areas (lanes 4–5). For the sCI63 protein, we observed a weak accumulation in the both areas for *gus*-siRNA, and strong accumulation mainly in del area for *gus*-mRNA (lanes 8–9). For the sMg1 protein, we observed a contrasting situation between the del and dis areas; lower accumulation of *gus*-si-RNA and accumulation of mRNA in the del area, and an absence of *gus*-mRNA in the dis area (Figure 6B, lanes 6–7). Then, for the same samples, we investigated the presence of P1 in the del and dis areas by western blot. Similar P1 protein accumulation was revealed in del and dis zones for sTz3 and sMg1 (Figure 6C, lanes 4–7), and only in the dis zone for sCI63 (Figure 6C, lanes 8–9). Although we cannot rule out the bombardment of P1 outside of GFP area, the strong accumulation of P1 detected in distal areas seems to favour the idea that of there being cell-to-cell movement from delivery to distal area of P1 proteins in rice leaves. This would help clarify our previous observation of GUS activity being detected across entire leaves (see Figure 5A, lanes 2 and 6 compared to 8 or 9). Furthermore, we observed differences in P1 accumulation as shown for sCI63 (only detected in the dis area, Figure 6C lanes 8–9), where a weak accumulation of *gus*-siRNA was correlated with *gus*-mRNA expression, as compared to levels observed for sTz3 and sMg1. It therefore seems likely for sCI63 that the faster movement of this P1 protein is associated with its high efficiency in silencing suppression.

In contrast, for sMg1 we observed a different situation with the accumulation of P1 in the dis area which was not correlated with a decrease in gus-siRNA and the expression of *gus*-mRNA (Fig 6B and 6C, lanes 6–7). For sMg1, although movement of P1 occurred its lack of effects on *gus*-siRNA was likely caused by its weaker efficiency in silencing suppression.

### Functional amino acids in P1 proteins

**Figure 7:**
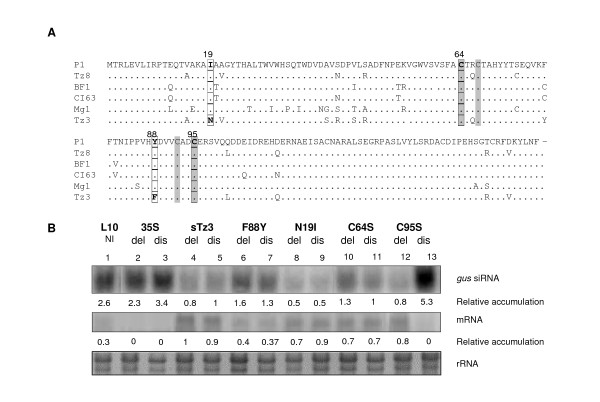
**Functional amino acids of P1 involved in silencing suppression**. (A) ClustalW alignment of P1 proteins with the putative Zn-finger (in grey) and detection of potential amino acids involved in silencing suppression that were point mutated (in box). (B) Northern analysis of *gus*-siRNA and mRNA at 2 days post delivery (dpd) with sTz3, and mutated sTz3 (F88Y, N19I, C64S and C95S) in the del or dis parts of the leaf (see Figure 5A). 35S is an empty cassette (negative control) and EtBr staining of rRNA served as a loading control. Relative accumulation to the loading control are shown, RNA band intensities were quantified using Image Quant software (Molecular Dynamics).

As we demonstrated major differences in the silencing suppression efficiencies of different P1 proteins (Tz8, BF1, CI63, Mg1 and Tz3), we attempted to assign amino acid diversity to functional effects with ClustalW alignments [36] (Figure 7A).

First, we identified the putative eukaryotic Zn-finger motif (C64(x)_2_C67(x)_24_C92(x)_2_C95) which is conserved across the RYMV phylogeny (Figure 7A). This kind of motif has been reported to be crucial for silencing suppression activity [37]. We introduced two independent single mutations in the Tz3 Zn-finger with PCR amplification in cysteine 64 and 95 (Cystein changed by Serine, C64S et C95S). Secondly, when comparing sequences of the strongest silencing suppressor (sTz3) and the weaker silencing suppressor (sMg1) we detected two amino acids specific to sTz3 (i.e. N19 and F88) (Figure 7A). We used PCR amplification to introduce two independent single mutations in the sTz3 sequence at asparagine 19 (Asparagine changed by Isoleucine, N19I) and phenylalanine 88 (Phenylalanine changed by Tyrosine, F88Y). The efficacy of these mutants to abolish silencing of the *uidA *transgene in L10 was tested following biolistic delivery. The effects of these mutations were compared with wild-type sTz3 and sMg1 silencing suppressor proteins.

At the histochemical and molecular levels (i.e. *gus*-specific si- and mRNA detection), we demonstrated that none of the four mutations completely abolished the suppression effect of sTz3. Nevertheless, the single amino acid mutation C95S impaired the ability of P1 to suppress *uid*A silencing in the distal part of leaves (high level of *gus*-siRNA), suggesting that this mutation affects the cell-to-cell movement of P1 (Figure 7B, lanes 12–13). Conversely, mutations F88Y or C64S both decreased silencing efficiency, as shown by the steady-state level of *gus*-siRNA in the del and dis zones being higher than observed with sTz3 (Figure 7B, lanes 4–7). Finally, mutation N19I did not affect the silencing suppression efficiency of the P1 protein relative to sTz3, as shown by similar pattern of *gus*-siRNA and *gus*-mRNA (Figure 7B, lanes 8–9). Together these results demonstrate that mutations highly influence the quantitative (Figure 7B, lanes 12–13) and qualitative (Figure 7B, lanes 6, 8, 10) expression of P1 activity. Finally, we found that the cystein 95 belonging to the putative zing finger is essential for the cell-to-cell movement of P1 and cystein 64 and phenylalanine 88 were involved in the efficiency of silencing suppression of the P1-Tz3 protein.

## Discussion

Previous studies have shown the potential of viral proteins as silencing suppressors, but only outside of their natural context [38]. For RYMV, the P1 protein was identified as a silencing suppressor in *N. benthamiana*, which is not a natural host [9]. The novelty of our study was the focus on the characterisation of silencing suppression with RYMV virions and the role of the P1 protein when infecting a natural host. We demonstrated the efficiency of the RYMV-virion as a constitutive-silencing suppressor in rice. We found for the first time that highly diverse RYMV isolates are all able to suppress a constitutive *gus*-silenced transgene, indicating that silencing suppression is a vital feature for RYMV. Through the quantitative analysis of GUS activity, we demonstrated that silencing suppression was dependent on viral presence in systemically infected leaves, but high silencing suppression is not strictly linked with a high viral content. Furthermore, we have also shown that the efficiency of GUS silencing reversion is isolate-dependent. Finally, we demonstrated that variation in silencing suppression was not linked to viral phylogeny and it occurred at the species and serogroup levels.

In order to explain the diversity observed at the virion scale, we focused on the P1 protein previously identified as a RYMV silencing suppressor [9]. Our results showed that the strength of silencing suppression at the virion scale was neither correlated with P1 protein accumulation, nor strictly correlated to P1 sequence variation. This complex picture at the virion scale suggests that the activity of the silencing suppressor protein is probably highly regulated during host-virus interactions. We cannot rule out the presence of additional silencing suppressor proteins encoded by RYMV, as has been shown for the *Citrus tristeza virus *[39], nor can we exclude the possibility of additional mechanisms known to overcome PTGS. For example, the *Brome mosaic virus *replicates its viral genome in endoplasmic reticulum where dsRNA is not accessible [40,41], and PVX has developed high-speed replication to outrun the mobile silencing signal [41].

Our observations have shown that silencing suppression efficacy and isolate pathogenicity were not correlated. This could contradict the hypothesis that silencing suppressors determine pathogenicity [42]. Although the basic hypothesis is that viral pathogenicity can be determined by the influence of a viral suppressor on miRNA pathways [43,44] it was recently demonstrated that not all silencing suppressors affect miRNA [45]. During infection in a natural context, suppressors are likely to be highly regulated so as to minimize their effect within the host. The silencing suppression effect of each RYMV isolate could thus be adapted to balance the viral effect (i.e. pathogenicity) in the host, while limiting viral-induced host gene silencing in order to preserve the integrity of the plant machinery during the infection process. RYMV also goes to different cytoplasmic organelles that could secondarily increase its pathogenicity [46]. This could mask the primary effect of the silencing suppressor. Interestingly, it is also known that some isolates can affect rice genotypes differently, suggesting that the silencing suppression state, as well as pathogenicity, could be genotype-dependent [27].

Our results, obtained with the P1 protein alone, showed a similar tendency in silencing suppression as to those obtained with virions suggesting a major role for the P1 silencing suppressor. As observed at the scale of the virion, we observed a distinct functional variation of P1 proteins based on the efficiency of silencing suppression, which was not correlated with the viral phylogeny.

We correlated variation in silencing suppression and sequence variation of protein P1 by molecular analyses, with independent point mutations in the Zn-finger motif or to selected amino acids identified by comparing sequence data between Tz3 (strongest suppressor) and Mg1 (lowest suppressor) P1 proteins. P1 mutants only showed partial alteration of the silencing suppression function. Cystein C95 has been shown to be essential for the cell-to-cell movement of protein P1, and C64, F88 essential for the strength of silencing. This contrasts with p19 from the *Tomato bushy stunt virus*, where a single mutation at G72 is sufficient to abolish the silencing suppression effect on PTGS [47]. These results indicate that silencing suppression might involve several domains in P1, as has been identified for P25 of PVX [35]. Based on the multifunctionality of viral proteins, such mutations could affect other roles involving movement or viral pathogenicity and which could be tested in rice with infectious full-length clones [48]. Mutation C95 was previously introduced in the full-length RYMV cDNA and it has been demonstrated that this full-length was not infectious (Bonneau and Brugidou, unpublished data). Here we demonstrated that this mutation affects cell-to-cell movement of the protein P1, and consequently, silencing suppression activity failed in neighbouring cells. At this point, we cannot conclude if the absence of infectivity was due to absence of viral genomic transport or silencing suppression. However, we demonstrated that P1 movement is necessarily required for the spread of silencing suppression.

## Conclusion

In its natural host we highlighted the functional diversity of RYMV isolates on silencing suppression. The functional diversity at the scale of virion is not correlated to genetic diversity of the P1 protein suppressor. In addition, we demonstrated that this functional diversity is not linked to the virus phylogeny or its pathogenicity. Our overall results suggest that the mechanism of silencing suppression due to the virus is more complex than previously thought. By studying the P1 silencing suppressor alone we demonstrated that the functional diversity of silencing suppression occurs at the P1 protein level and mutagenesis experiments demonstrated that cystein C95 is essential for cell-to-cell movement of P1, and that C64 and F88 are involved in the efficiency of silencing suppression. As both cysteins (C95 and C64) are conserved in the RYMV phylogeny, and as F88 is not, we could assume that this last mutation could be one of the amino acid involved in silencing suppression diversity of P1. This mutation opens the way to identify other amino acids involved in the functional diversity of silencing suppression.

## Abbreviations

RYMV: *rice yellow mottle virus*; dsRNA: double strand RNA; miRNA: microRNA; siRNA: small-interfering RNA; PTGS: post-transcriptional gene silencing; ORF: open reading frame; GUS: β-glucoronidase; *uidA*: GUS gene; cv: cultivar; O.s: *Oryza sativa*; TAI: Taipei; BF: Burkina Faso; CI: Ivory Coast; Mg: Madagascar; Ni: Nigeria; Tz: Tanzania; DAS-Elisa: double antibody sandwich-enzyme linked immunosorbent assay; UTR: untranslated region; CaMV: *cauliflower mosaic virus*; CP: coat protein; GFP: Green fluorescent protein; PVX: *potato potexvirus *; EtBr: ethidium bromide; NI: non-inoculated; BI: buffer-inoculated, ND: non-biolistic delivery; BD: biolistic delivery; dpd: day post delivery; del: delivery; dis, distal.

## Competing interests

The authors declare that they have no competing interests.

## Authors' contributions

CS carried out experiments and drafted the manuscript. MB-R participated in the design, cloning, mutagenesis and transitory expression assays; DF participated in the design of experiments in Figs 2, 3, 4 and table 1; CB conceived this project, participated in the study design and coordination and helped to draft the manuscript. All authors read and approved the final manuscript.
